# Virtual-Integrated Admittance Control Method of Continuum Robot for Capturing Non-Cooperative Space Targets

**DOI:** 10.3390/biomimetics10050281

**Published:** 2025-04-30

**Authors:** Lihua Wang, Zezhou Sun, Yaobing Wang, Jie Wang, Chuliang Yan

**Affiliations:** 1 School of Mechanical and Aerospace Engineering, Jilin University, Changchun 130025, China; wlh20@mails.jlu.edu.cn (L.W.);; 2 Beijing Institute of Spacecraft System Engineering, Beijing 100094, China

**Keywords:** continuum robots, whole-arm grasping, admittance control, potential field

## Abstract

Continuum robots (CRs) are highly effective in grasping moving targets in space through whole-arm grasping (WAG), offering broad applicability and reliable capture. These characteristics make CRs particularly suitable for capturing non-cooperative space targets. Compliant control plays a crucial role in ensuring safe and reliable interactions during the grasping process. This paper proposes a virtual-integrated admittance control (VIAC) method specifically designed to enhance WAG by CRs. By proactively adjusting the robot’s trajectory before contact, the VIAC method effectively reduces the contact force exerted on the target during grasping, enabling compliant capture while preventing target escape and minimizing potential damage. This study first develops a mathematical model of the CR and addresses the inverse dynamics problem. Subsequently, the VIAC method is introduced to regulate contact force and improve grasping performance. This approach integrates virtual forces, derived from position information, with actual contact forces acting on the robot’s links, facilitating trajectory replanning through an admittance controller. The virtual forces, constructed based on improved virtual potential fields, reduce the relative velocities of robot links with respect to the target during the approach, ensuring successful grasping. Simulation results validate the effectiveness of the VIAC method, demonstrating a significant reduction in contact force compared to conventional admittance control.

## 1. Introduction

The escalating accumulation of space debris poses a substantial threat to operational spacecrafts, occupying limited orbital resources and undermining the long-term sustainability of the space environment. Active debris removal (ADR) has been proposed as a viable solution to mitigate these risks and maintain orbital stability [1,2]. Existing ADR methodologies can be broadly categorized into two primary approaches: one employing robotic arms [3,4,5,6], capture nets [7,8,9,10], harpoons [11,12,13,14], tether systems [15,16,17], and flexible mechanisms [18,19,20,21] to capture and deorbit debris; and another utilizing lasers [22,23,24,25] or ion beams [26,27,28] to push debris out of orbit. Among these, the robotic arm-based approach has gained significant attention due to its technological maturity and reliability [29]. Nevertheless, conventional robotic arms, constrained by their limited degrees of freedom, encounter challenges in fulfilling the demands of adaptable grasping in complex space environments. Furthermore, single-point grasping requires precise target identification and increases the probability of grasping failure due to stress concentration.

To overcome these limitations, recent research has explored the application of continuum robots (CRs) for capturing non-cooperative space targets. Inspired by biological structures such as elephant trunks and octopus tentacles, CRs exhibit a high degree of flexibility and numerous degrees of freedom, making them well suited for unstructured environments [30]. CR-based capture strategies can be classified into two primary methodologies: (1) end-effector grasping, which employs a claw or manipulator mounted at the robot’s end; and (2) whole-arm grasping (WAG), wherein the robot wraps around the target with its entire body. Compared to end-effector grasping, WAG provides greater error tolerance. Additionally, the distribution of contact forces across multiple points mitigates stress concentration and improves capture success rates. These capabilities are particularly advantageous for ADR missions. For example, CRs can be deployed to softly capture large, slowly tumbling satellites in geostationary orbit that have lost attitude control, without the need for precise grasping point detection. Similarly, they are promising for deorbiting damaged solar panel assemblies or fuel tank debris in low Earth orbit.

Recent studies have explored grasping configurations and compliant control strategies for CRs in grasping tasks. Peng et al. [31] proposed a cooperative planning method for multi-arm space robots, optimizing joint equivalent torque during pre-capture and post-capture phases under constraints such as joint angles, cable tension, and end-effector velocity. Li et al. [32] investigated a force–position collaborative optimization method for capturing non-cooperative space targets using rope-driven snake manipulators equipped with end-effectors. Their approach optimized joint motion and active stiffness through a force–position collaborative index, achieving a 25% reduction in contact force compared to conventional impedance control. Jiang et al. [33] developed a real-time integrated controller for dual-arm continuum manipulators in space capture missions, utilizing the MATD3 algorithm for motion planning and collision avoidance. However, these studies primarily focus on end-effector grasping, which requires the precise tracking of specific grasping points, thereby limiting task flexibility. In contrast, WAG eliminates the need for precise grasping point tracking and accommodates a broader range of shape variations. Li et al. [34,35,36] conducted extensive research on grasping configurations and continuum grasping, performing comprehensive experiments with OctArm. Wilde et al. [37] developed a tentacle-inspired CR and performed experiments to capture a drifting and rotating target under microgravity conditions. Liu et al. [38] introduced an elephant trunk robot driven by a single motor, designed to adapt to target shapes while considering extreme environmental constraints. Li et al. [39] simulated the detumbling of a target using a space robot equipped with four pneumatic flexible arms. Agabiti et al. [40] proposed two WAG strategies based on reinforcement learning and finite element simulation. Numerous studies have examined diverse CR architectures [41,42,43,44,45], with experimental results demonstrating their ability to maneuver flexibly and perform WAG effectively.

In the context of compliant control, impedance control and admittance control are two widely used methods, both aiming to regulate contact forces indirectly by modulating the dynamic equilibrium between the robot and external forces. Li et al. [46] designed an indirect force sensor for a cable-driven redundant manipulator, integrating cable tension sensors and encoders to facilitate admittance control. Xia et al. [47] proposed a capture strategy for targets of varying dimensions, employing an adaptive impedance control approach to ensure stable and secure grasping. Su et al. [48] introduced a multi-point coordinated variable impedance control scheme, analyzing the stability of both the external load estimator and the controller. However, impedance control often necessitates high stiffness to resist external forces, requiring the rapid delivery of substantial torque from the robot’s joints; admittance control adjusts displacement in response to external forces, which may potentially lead to excessive instantaneous acceleration if the external force is significant. Non-contact impedance control regulates the relative motion between the robot and the target using the robot’s kinematic state through a virtual impedance model, even in the absence of physical contact. This method enhances performance in obstacle avoidance and contact-sensitive tasks. Ding et al. [49] introduced a non-contact impedance control-based obstacle avoidance methodology for mobile robots, improving motion smoothness during local autonomous navigation. Arita et al. [50] proposed a serial combination control strategy, integrating non-contact impedance control with force control to mitigate impact force and facilitate smooth contact transitions.

The aforementioned research highlights the distinct advantages of WAG with CRs for capturing non-cooperative space targets, underscoring its practical engineering significance. However, existing studies primarily focus on structural design and motion planning, with limited exploration of control strategies and their effectiveness. While some research addresses control, the emphasis is often on lightweight objects in terrestrial environments, leaving a notable gap in the study of compliant control for WAG in space applications. To address this gap, this paper proposes a virtual-integrated admittance control (VIAC) method. The term “virtual-integrated” refers to the enhancement of a conventional admittance control framework by incorporating virtual forces. These virtual forces are not treated separately but are integrated with physical contact forces, allowing proactive motion adjustments through a unified control law. Specifically, this approach introduces virtual forces based on position information within the admittance control framework, enabling proactive trajectory adjustments as the CR’s links approach the target to reduce impact forces upon contact. After contact, the robot’s trajectory is further refined based on actual contact forces, ensuring compliance with external forces and facilitating successful grasping. Unlike prior hybrid or virtual force-based methods, the VIAC method unifies virtual repulsive and guiding forces with contact force feedback within a single admittance control framework, enabling smooth transitions and supporting distributed compliance along the entire arm, which is essential for WAG tasks. Simulation results validate the effectiveness of the VIAC method, demonstrating a significant reduction in contact forces during grasping while enhancing the dynamic performance of the CR.

The remainder of this paper is organized as follows: Section 2 presents the CR’s structure, along with its kinematic and dynamic models; Section 3 details the compliant control method for WAG; Section 4 presents simulation results validating the VIAC method; and Section 5 concludes this paper and outlines future research directions.

## 2. Modeling

### 2.1. Mechanism Design

**Figure 1:**
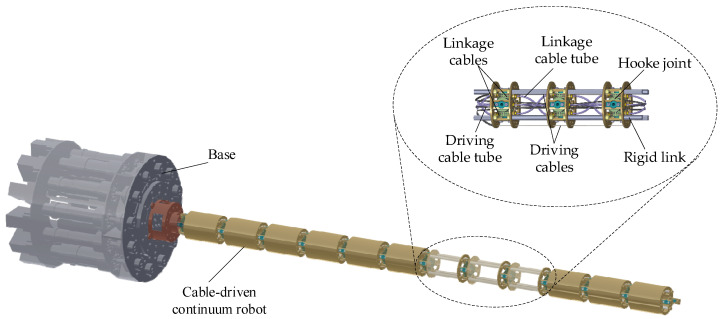
Mechanical design of cable-driven CR.

As illustrated in Figure 1, an underactuated CR with decoupled driving cables for each segment is presented [51]. The cable-driven CR consists of $n_{ss}$ segments, with each segment comprising $n_{ss}$ links, $n_{ss}$ Hooke joints, three driving cables, $n_{ss}-1$ sets of linkage cables, and their corresponding driving and linkage cable tubes. Each set of linkage cables ensures synchronized motion between adjacent Hooke joints within a segment, while the driving cable tubes mechanically decouple the driving cables of one segment from those of another. The entire driving system, including motors, winding drums, and redirecting pulleys, is housed within the base, separated from the joints. This structure design significantly reduces the weight of the robotic arm, enhancing its efficiency for space applications. Additionally, its modular architecture enables task-specific customization, facilitates system expansion, and enhances adaptability across diverse operational scenarios.

### 2.2. Kinematic Modeling

**Figure 2:**
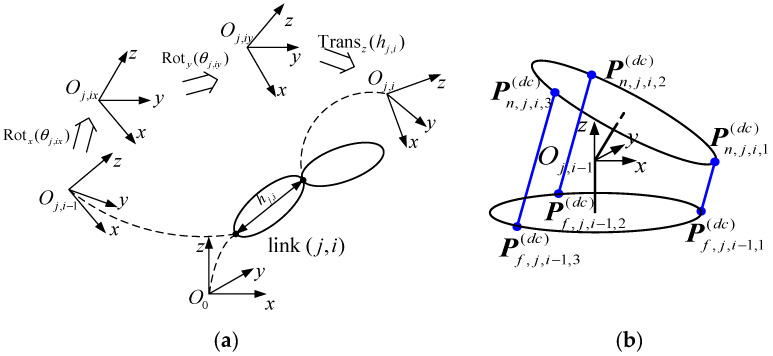
Kinematic relationships of CR between different spaces: (**a**) Joint-task space. (**b**) Joint-driving space.

The CR described above exhibits a highly modular architecture, comprising multiple identical links connected in series to form individual segments, which are further connected in series to construct the complete robotic system. To establish the kinematic model, this section considers a single Hooke joint as an illustrative example. The kinematic relationships of the CR are formulated across driving space, joint space, and task space. As illustrated in Figure 2, this section outlines the transformation relationships within the CR across joint-task space and joint-driving space, which are further detailed in the subsequent subsections.

#### 2.2.1. Joint-Task Space

To describe the pose of the CR in Cartesian space, coordinate frames are assigned to the end of each link. As illustrated in Figure 2a, frames {j,i−1} and {j,i} are defined. Specifically, frame {j,i−1} is established at the end of link i−1 in segment j and also serves as the initial frame for joint i of segment j $\left(j=1,\;2,\cdots ,n_{s};\;i=1,\;2,\;\cdots ,n_{ss}\right)$. The homogeneous transformation matrix from frame {j,i−1} to frame {j,i} is expressed as

(1) Tj,ij,i−1=Rotxθj,ixRotyθj,iyTranszhj,i

where $\theta_{j,ix}$ and $\theta_{j,iy}$ denote the rotation angles of joint (j,i) with respect to frame {j,i−1} around axis $x_{j,i-1}$ and to frame {j,ix} around axis $y_{j,ix}$, respectively, as depicted in Figure 2a; $h_{j,i}$ denotes the length of link (j,i). Within segment j, due to the effect of the linkage cables, the rotation angles in the same direction remain equal for all joints, such that $\theta_{j,ix}=q_{jx}$, $\theta_{j,iy}=q_{jy}$.

The homogeneous transformation matrix from the initial frame to the end frame of segment j can be obtained as follows:

(2) Tjj−1=Tj,1j,0Tj,2j,1⋯Tj,nssj,nss−1

where Tj,ij,0=Tj,ij−1,nss, indicating that the frame at the start of segment j coincides with the end frame of segment j−1.

Consequently, the homogeneous transformation matrix from the base frame to the end-effector of the CR is expressed as follows:

(3) Tnsb=T1bT21⋯Tnsns−1

where T1b represents the homogeneous transformation matrix from the base frame to the frame $\left\{1,1\right\}$.

#### 2.2.2. Joint-Driving Space

The joint rotation is actuated by the combined effect of driving cables. As illustrated in Figure 2b, the driving cable $C_{j,k}^{\left(dc\right)}\;(k=1,2,3)$ of joint (j,i) passes through the cable hole $P_{f,j,i,k}^{\left(dc\right)}$ on disk $D_{f,j,i-1}$ of link (j,i−1) and the cable hole $P_{n,j,i,k}^{\left(dc\right)}$ on disk $D_{n,j,i}$ of link (j,i). The length of the driving cable $C_{j,k}^{\left(dc\right)}$ between the two disks is expressed as follows:

(4) lj,i,k(dc)=Pn,j,i,k(dc)j,i−1−Pf,j,i−1,k(dc)j,i−1

where $P_{n,j,i,k}^{\left(dc\right)}$ and $P_{f,j,i,k}^{\left(dc\right)}$ are given by the following:

(5) Pn,j,i,k(dc)j,i=[rj(dc)cos((k−1)2π3)rj(dc)sin((k−1)2π3)h0]TPf,j,i,k(dc)j,i=[rj(dc)cos((k−1)2π3)rj(dc)sin((k−1)2π3)hj,i−h0]T

where $r_{j}^{\left(dc\right)}$ is the radius of the circular path along which the cable holes are evenly distributed within segment j, and $h_{0}$ is the distance from disk $D_{n,j,i}$ to the geometric center of the joint (j,i).

The length of the driving cables in segment j is as follows:

(6) $$L_{j,k}^{\left(dc\right)}=\sum_{i=1}^{n_{ss}}l_{j,i,k}^{\left(dc\right)}+\sum_{i=1}^{n_{ss}-1}h_{j,i}$$

### 2.3. Differential Kinematics

#### 2.3.1. Joint-Task Space

The Jacobian matrix between the joint space and the task space characterizes the velocity mapping relationship between the two spaces. The velocity of point P on the backbone of the CR can be expressed in terms of the joint velocities as follows:

(7) $$\dot{P}=\left[\begin{array}{c} \upsilon \\ \omega \end{array}\right]=J_{x-q}\dot{Q}$$

where $Q\in R^{2n_{s}\times 1}$ is the column vector of joint variables, and $J_{x-q}\in R^{6\times 2n_{s}}$ is the Jacobian matrix mapping joint velocities to Cartesian velocities.

#### 2.3.2. Joint-Driving Space

The Jacobian matrix between the joint space and the driving space establishes the relationship between joint velocities and driving cable velocities. The relationship is given by the following:

(8) $$\dot{L}=J_{l-q}\dot{Q}$$

where $\dot{L}\in R^{3n_{s}\times 1}$ is a vector composed by driving cable velocities components for all segments, and $J_{l-q}\in R^{3n_{s}\times 2n_{s}}$ is the Jacobian matrix mapping joint velocities to driving cable velocities.

Since the CR described in this study features decoupled actuation, the rate of change in the length of the driving cables is only dependent on the joint angles of the segment that they actuate, without interference from other segments.

### 2.4. Dynamic Modeling

This section analyzes the dynamics of the CR based on the following assumptions:

(1) The friction between the driving cable tubes and driving cables, as well as between the linkage cable tubes and linkage cables, is negligible;
(2) The resistance of the driving cable tubes to backbone bending is negligible;
(3) The fit between the driving cables and the cable holes is seamless, and the linkage cables do not slip;
(4) The cables are considered non-deformable.

These ideal assumptions above are valid for enabling tractable dynamic modeling. However, during fast dynamic maneuvers or under high external loads, non-negligible friction or cable elasticity may introduce energy loss and reduce motion precision. Similarly, if the linkage cables slip or deform, the accuracy of joint coordination may degrade. These effects could influence the dynamic behavior of the CR and would need to be compensated for through closed-loop control strategies.

A force analysis is conducted on links at different positions of the CR. Based on the robot’s structure, the forces acting on each link include the following: linkage cable force $F^{\left(lc\right)}$, driving cable force $F^{\left(dc\right)}$, interaction force between adjacent links $F^{\left(al\right)}$, inertial force $F^{\left(ci\right)}$, and external force $F^{\left(ext\right)}$. Note that F here represents the generalized force vector, $F={\left[\begin{array}{cc} f & m \end{array}\right]}^{T}\in R^{6\times 1}$.

**Figure 3:**
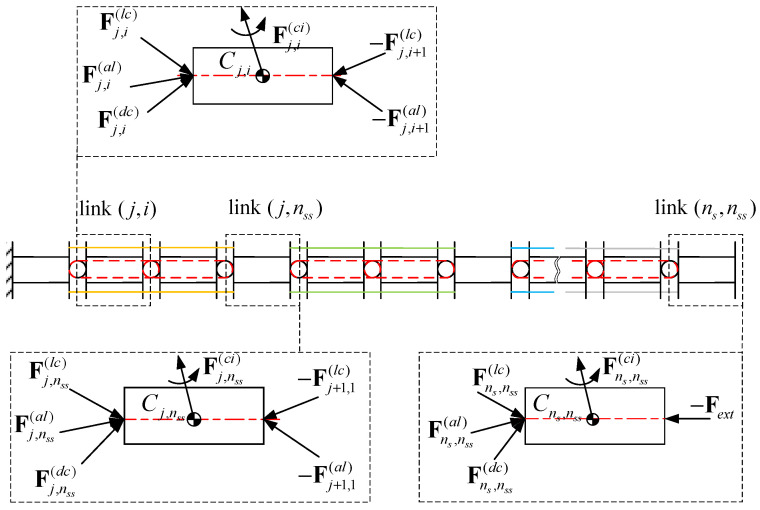
Force conditions of links at different positions of the CR.

Figure 3 illustrates the force conditions of links at different positions of the CR. The end link $\left(n_{s},\;n_{ss}\right)$, is not subject to reaction force from a subsequent link nor coupled with a subsequent joint but is subject to $-F^{\left(ext\right)}$. The link (j, n_ss) is subject to interaction forces and linkage cable forces from adjacent links, while the driving cable forces originate from different segments. The link (j, i) is subject to forces originating from the same segment. The force balance equation at the center of mass $C_{j,i}$ of the link is as follows:

(9) $$F_{j,i}^{\left(al\right)}+F_{j,i}^{\left(lc\right)}+F_{j,i}^{\left(dc\right)}-F_{j,i+1}^{\left(al\right)}-F_{j,i+1}^{\left(lc\right)}-F_{j,i}^{\left(ext\right)}-F_{j,i}^{\left(ci\right)}=0$$

The equivalent interaction force is defined as ${\tilde{F}}_{j,i}^{\left(al\right)}=F_{j,i}^{\left(al\right)}+F_{j,i}^{\left(lc\right)}$; thus, Equation (9) can be rewritten as follows:

(10) $$F_{j,i}^{\left(ci\right)}={\tilde{F}}_{j,i}^{\left(al\right)}-{\tilde{F}}_{j,i+1}^{\left(al\right)}+F_{j,i}^{\left(dc\right)}-F_{j,i}^{\left(ext\right)}$$

where ${\tilde{F}}_{j,i+1}^{\left(al\right)}=0$ for link $\left(n_{s},n_{ss}\right)$, and ${\tilde{F}}_{j,i+1}^{\left(al\right)}={\tilde{F}}_{j+1,1}^{\left(al\right)}$ for link $\left(j,n_{ss}\right)$ $\left(j=1,2,\ldots ,n_{s}-1\right)$.

A systematic analysis of the driving cable force $F^{\left(dc\right)}$, linkage cable force $F^{\left(lc\right)}$, and inertial force $F^{\left(ci\right)}$ is conducted in the subsequent sections. Subsequently, the recursive dynamic equations of the CR are derived based on the force equilibrium expressions.

#### 2.4.1. Driving Cable Force $F^{\left(dc\right)}$

As outlined in the previous structural description, each segment of the CR is driven by three driving cables. The driving cables of segment j, encapsulated within driving cable tubes, pass through the preceding j−1 segments. This design ensures that the motion of each segment is decoupled, relying solely on its own driving cables without interference from adjacent segments. Specifically, the proximal ends of driving cables for segment j are anchored to disk $D_{f}$ on link $\left(j-1,n_{ss}\right)$, while their distal ends are attached to disk $D_{n}$ on link $\left(j,n_{ss}\right)$.

**Figure 4:**
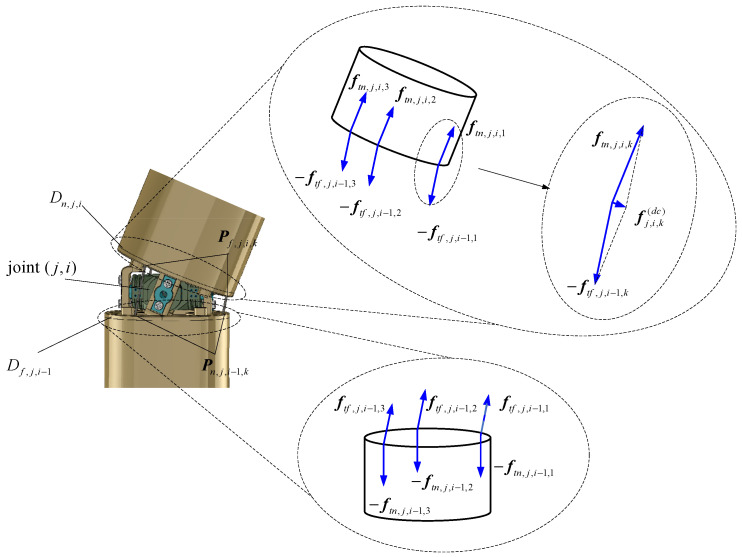
Diagram of driving cable forces.

Figure 4 depicts the forces exerted by the driving cables on disks $D_{n,j,i}$ and $D_{f,j,i-1}$, which are connected by joint (j,i). The resultant forces at the cable holes $P_{f,j,i,k}$ and $P_{n,j,i,k}$ $\left(j=1,2,\cdots ,n_{s};i=1,2,\cdots ,n_{ss};k=1,2,3\right)$ are represented as $f_{f,j,i,k}^{\left(dc\right)}$ and $f_{n,j,i,k}^{\left(dc\right)}$, respectively.

$f_{f,j,i,k}^{\left(dc\right)}$ and $f_{n,j,i,k}^{\left(dc\right)}$ are given by the following:

(11) $$f_{f,j,i,k}^{\left(dc\right)}=\left\{\begin{array}{ll} f_{tf,j,i,k}-f_{tn,j,i,k}, & 1\leq j\leq n_{s},1\leq i\leq n_{ss}-1\\ f_{tf,j,i,k}, & 1\leq j\leq n_{s}-1,i=n_{ss}\\ 0, & j=n_{s},i=n_{ss} \end{array}\right.$$

(12) $$f_{n,j,i,k}^{\left(dc\right)}=\left\{\begin{array}{ll} f_{tn,j,i,k}-f_{tf,j,i-1,k}, & 1<j\leq n_{s},1<i\leq n_{ss}-1\\ -f_{tf,j,i-1,k}, & 1\leq j\leq n_{s},i=n_{ss}\\ f_{tn,j,i,k}-f_{tf,base,k}, & j=1,i=1 \end{array}\right.$$

where $f_{tf,j,i,k}$ and $f_{tn,j,i,k}$ represent the driving cable forces shown in Figure 4, and $f_{tf,base,k}$ represents the driving cable force at the base. They are calculated as follows:

(13) $$f_{tf,j,i,k}=\left\{\begin{array}{ll} f_{j,k}^{\left(dc\right)}e_{tf,j,i,k}, & 1\leq j\leq n_{s},1\leq i\leq n_{ss}-1\\ f_{j+1,k}^{\left(dc\right)}e_{tf,j,i,k}, & 1\leq j\leq n_{s}-1,i=n_{ss} \end{array}\right.$$

(14) $$f_{tn,j,i,k}=f_{j,k}^{\left(dc\right)}e_{tn,j,i,k}$$

(15) $$f_{tf,base,k}=f_{1,k}^{\left(dc\right)}e_{tf,base,k}$$

where $f_{j,k}^{\left(dc\right)}$ is the tension of the kth driving cable in segment j, and etf,j,i,kj,i, etn,j,i,kj,i and etf,base,kj,i are the direction vectors expressed in the local frame $\left\{j,i\right\}$, computed as

(16) etf,j,i,kj,i=Pn,j,i+1,kj,i−Pf,j,i,kj,iPn,j,i+1,kj,i−Pf,j,i,kj,i,(j=1,2,⋯,ns−1;i=1,2,⋯,nss−1)Pn,j+1,1,kj,i−Pf,j,i,kj,iPn,j+1,1,kj,i−Pf,j,i,kj,i,(j=1,2,⋯,ns−1;i=nss)

(17) etn,j,i,kj,i=Pf,j,i,kj,i−Pn,j,i,kj,iPf,j,i,kj,i−Pn,j,i,kj,i,(j=1,2,⋯,ns;i=1,2,⋯,nss)Pf,j,i,kj,i−Pn,j−1,nss,kj,iPf,j,i,kj,i−Pn,j−1,nss,kj,i,(j=1,2,⋯,ns;i=1)

(18) etf,base,kj,i=Ptn,1,1,kj,i−Ptf,base,kj,iPtn,1,1,kj,i−Ptf,base,kj,i

The torque generated by transferring the driving cable force from the cable hole to the centroid of the link can be obtained:

(19) $$n_{f,j,i,k}^{\left(dc\right)}=r_{f,j,i,k}^{\left(c\right)}\times f_{f,j,i,k}^{\left(dc\right)}=(P_{f,j,i,k}-O_{j,i})\times f_{f,j,i,k}^{\left(dc\right)}$$

(20) $$n_{n,j,i,k}^{\left(dc\right)}=r_{n,j,i,k}^{\left(c\right)}\times f_{n,j,i,k}^{\left(dc\right)}=(P_{n,j,i,k}-O_{j,i})\times f_{n,j,i,k}^{\left(dc\right)}$$

where $O_{j,i}$ is the origin of frame {j,i}.

Thus, the generalized driving cable force can be obtained:

(21) $$F_{j,i}^{\left(dc\right)}=\sum_{k=1}^{3}\left[\begin{array}{c} f_{j,i,k}^{\left(dc\right)}\\ n_{j,i,k}^{\left(dc\right)} \end{array}\right]$$

#### 2.4.2. Linkage Cable Force $F^{\left(lc\right)}$

**Figure 5:**
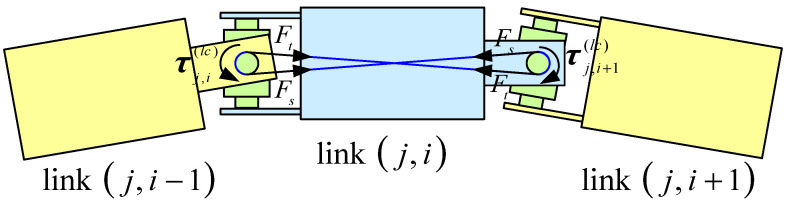
Schematic diagram of the linkage mechanism.

The motion of adjacent joints in the same direction is synchronized by a set of linkage cables, forming a figure-eight configuration between two consecutive joints. The schematic diagram of the equivalent linkage mechanism is illustrated in Figure 5:

Initially, the linkage cables on both sides are pre-tensioned with an identical initial pre-tension. When the robot moves under external forces, the tension distribution in the linkage cables changes. As illustrated in Figure 5, the tension decreases in the slack-side cable while increasing in the tight-side cable. The equivalent torques generated by the linkage cables are calculated as follows:

(22) $$\left\{\begin{array}{l} \tau_{j,i}^{\left(lc\right)}=r\left(F_{t}-F_{s}\right)\\ \tau_{j,i+1}^{\left(lc\right)}=-r\left(F_{t}-F_{s}\right) \end{array}\right.$$

where r represents the winding radius of the linkage cable. The equivalent torques exerted at adjacent joints by the same set of linkage cables are equal in magnitude but opposite in direction.

Since all joints within each segment are passive, the torque between adjacent links is equal to the equivalent torque generated by the linkage cables. The equivalent torques $\tau_{j,i}^{\left(lc\right)}$ and $\tau_{j,i+1}^{\left(lc\right)}$ at joints (j,i) and (j,i+1) represent the torques from link (j,i) acting on links (j,i−1) and (j,i+1), respectively. It is important to note that, within segment j, link (j,1) has no preceding link connected, and link $\left(j,n_{ss}\right)$ has no subsequent link connected. Thus, the equivalent torques due to linkage cables within segment j satisfy

(23) $$n_{j,i}^{\left(lc\right)}=\left\{\begin{array}{l} \begin{array}{lll} {}[-n_{j,(i+1)x}^{\left(lc\right)} & -n_{j,(i+1)y}^{\left(lc\right)} & 0{]}^{T},i=1 \end{array}\\ \begin{array}{lll} {}[n_{j,ix}^{\left(lc\right)}-n_{j,(i+1)x}^{\left(lc\right)} & n_{j,iy}^{\left(lc\right)}-n_{j,(i+1)y}^{\left(lc\right)} & 0{]}^{T},2\leq i\leq n_{ss}-1 \end{array}\\ \begin{array}{lll} {}[n_{j,ix}^{\left(lc\right)} & n_{j,iy}^{\left(lc\right)} & 0{]}^{T},i=n_{ss} \end{array} \end{array}\right.$$

where $n_{j,ix}^{\left(lc\right)}$ and $n_{j,iy}^{\left(lc\right)}$ are the components of the equivalent torque at joint (j,i) along the x-axis and y-axis, respectively.

From the above analysis, the effect of the linkage cables can be equivalently represented as the interaction torques between connected links, wherein joints exhibit active motion capabilities. Therefore, the equivalent interaction torques between connected links must satisfy the following constraints:

(24) $$\left\{\begin{array}{l} n_{j,ix}^{{\left(al\right)}^{\prime }}=n_{j,ix}^{\left(lc\right)}\\ n_{j,iy}^{{\left(al\right)}^{\prime }}=n_{j,iy}^{\left(lc\right)} \end{array}\right.$$

#### 2.4.3. Inertial Force $F^{\left(ci\right)}$

To determine the inertial force, an outward iteration is performed to sequentially calculate the angular velocity, angular acceleration, linear acceleration, and centroid acceleration of each link. These are expressed as follows:

(25) $$\omega_{j,i}=\left\{\begin{array}{l} X_{1,1x}{\dot{\theta }}_{1,1x}+Y_{1,1y}{\dot{\theta }}_{1,1y},ji=1\\ \omega_{j,i-1}+X_{j,ix}{\dot{\theta }}_{j,ix}+Y_{j,iy}{\dot{\theta }}_{j,iy},ji>1 \end{array}\right.$$

(26) $${\dot{\omega }}_{j,i}=\left\{\begin{array}{l} X_{1,1x}{\ddot{\theta }}_{1,1x}+X_{1,1x}{\dot{\theta }}_{1,1x}\times Y_{1,1y}{\dot{\theta }}_{1,1y}+Y_{1,1y}{\ddot{\theta }}_{1,1y},ji=1\\ {\dot{\omega }}_{j,i-1}+\omega_{j,i-1}\times X_{j,ix}{\dot{\theta }}_{j,ix}+X_{j,ix}{\ddot{\theta }}_{j,ix}+(\omega_{j,i-1}+X_{j,ix}{\dot{\theta }}_{j,ix})\times Y_{j,iy}{\dot{\theta }}_{j,iy}+Y_{j,iy}{\ddot{\theta }}_{j,iy},ji>1 \end{array}\right.$$

(27) $${\dot{\upsilon }}_{j,i}=\left\{\begin{array}{l} {\left[\begin{array}{ccc} 0 & 0 & g \end{array}\right]}^{T},ji=1\\ {\dot{\upsilon }}_{j,i-1}+{\dot{\omega }}_{j,i-1}\times r_{j,i-1}+\omega_{j,i-1}\times (\omega_{j,i-1}\times r_{j,i-1}),ji>1 \end{array}\right.$$

(28) $${\dot{\upsilon }}_{j,i}^{\left(ci\right)}={\dot{\upsilon }}_{j,i}+{\dot{\omega }}_{j,i}\times r_{j,i}^{\left(ci\right)}+\omega_{j,i}\times (\omega_{j,i}\times r_{j,i}^{\left(ci\right)})$$

where $\omega_{j,i}$ is the angular velocity of link (j,i), ${\dot{\omega }}_{j,i}$ is the angular acceleration of link (j,i), ${\dot{\upsilon }}_{j,i}$ is the linear acceleration at the center of the joint (j,i), and ${\dot{\upsilon }}_{j,i}^{\left(ci\right)}$ is the linear acceleration at the centroid of link (j,i).

Based on the outward iteration results, the generalized inertial force $F_{j,i}^{\left(ci\right)}$ is derived from Newton’s and Euler’s equations:

(29) $$F_{j,i}^{\left(ci\right)}=\left[\begin{array}{c} f_{j,i}^{\left(ci\right)}\\ n_{j,i}^{\left(ci\right)} \end{array}\right]=\left[\begin{array}{c} m_{j,i}{\dot{\upsilon }}_{j,i}^{\left(ci\right)}\\ I_{j,i}^{\left(ci\right)}{\dot{\omega }}_{j,i}+\omega_{j,i}\times \left(I_{j,i}^{\left(ci\right)}\omega_{j,i}\right) \end{array}\right]$$

where $m_{j,i}$ represents the mass of link (j,i), and $I_{j,i}^{\left(ci\right)}$ represents the inertial tensor matrix of the rigid body to its center of mass.

#### 2.4.4. Recursive Dynamics

By incorporating the previously analyzed forces into Equation (10), the system dynamics can be expressed as follows:

(30) $$\left[\begin{array}{cc} \begin{array}{c} E\\ r_{j,i}^{\left(ci\right)}\times \end{array} & \begin{array}{c} 0\\ E \end{array} \end{array}\right]F_{j,i}^{\left(ci\right)}={\tilde{F}}_{j,i}^{\left(al\right)}-\left[\begin{array}{cc} \begin{array}{c} E\\ r_{j,i}\times \end{array} & \begin{array}{c} 0\\ E \end{array} \end{array}\right]{\tilde{F}}_{j,i+1}^{\left(al\right)}+F_{j,i}^{\left(dc\right)}-F_{j,i}^{\left(ext\right)}$$

Let $A_{j,i}=\left[\begin{array}{cc} E & 0\\ r_{j,i}\times & E \end{array}\right]$, $A_{j,i}^{\left(ci\right)}=\left[\begin{array}{cc} E & 0\\ r_{j,i}^{\left(ci\right)}\times & E \end{array}\right]$, and the equation above can be rewritten as

(31) $${\tilde{F}}_{j,i}^{\left(al\right)}=A_{j,i}{\tilde{F}}_{j,i+1}^{\left(al\right)}+A_{j,i}^{\left(ci\right)}F_{j,i}^{\left(ci\right)}-F_{j,i}^{\left(dc\right)}+F_{j,i}^{\left(ext\right)}$$

For segment $n_{s}$, the end link $\left(n_{s},n_{ss}\right)$ is not subjected to reaction force from a subsequent link. Therefore, the generalized equivalent interaction force acting on link $\left(n_{s},n_{ss}\right)$ is the following:

(32) $${\tilde{F}}_{n_{s},n_{ss}}^{\left(al\right)}=A_{n_{s},n_{ss}}^{\left(ci\right)}F_{n_{s},n_{ss}}^{\left(ci\right)}-F_{n_{s},n_{ss}}^{\left(dc\right)}+F_{n_{s},n_{ss}}^{\left(ext\right)}$$

Similarly, the generalized equivalent interaction force acting on link $\left(n_{s},i\right)$ is the following:

(33) $$\begin{array}{cc} {\tilde{F}}_{n_{s},i}^{\left(al\right)} & =A_{n_{s},i}{\tilde{F}}_{n_{s},i+1}^{\left(al\right)}+A_{n_{s},i}^{\left(ci\right)}F_{n_{s},i}^{\left(ci\right)}-F_{n_{s},i}^{\left(dc\right)}+F_{n_{s},i}^{\left(ext\right)}\\ & =\sum_{a=i}^{n_{ss}}\left(\left(\prod_{b=i+1}^{a}A_{n_{s},b-1}\right)A_{n_{s},a}^{\left(ci\right)}F_{n_{s},a}^{\left(ci\right)}\right)-\sum_{a=i}^{n_{ss}}\left(\left(\overset{a}{\underset{b=i+1}{\prod }}A_{n_{s},b-1}\right)F_{n_{s},a}^{\left(dc\right)}\right)+\sum_{a=i}^{n_{ss}}\left(\left(\overset{a}{\underset{b=i+1}{\prod }}A_{n_{s},b-1}\right)F_{n_{s},a}^{\left(ext\right)}\right) \end{array}$$

Based on the analysis in Section 2.4.2, the constraint conditions for segment $n_{s}$ can be expressed as follows:

(34) $$\sum_{i=1}^{n_{ss}}A_{\tau }R_{n_{s},i}^{-1}{\tilde{F}}_{n_{s},i}^{\left(al\right)}=0^{6\times 1}$$

where $A_{\tau }$ is an auxiliary matrix, $A_{\tau }=\mathrm{diag}(\left[\begin{array}{cccccc} 0 & 0 & 0 & 1 & 1 & 0 \end{array}\right])$, and $R_{n_{s},i}\in R^{3\times 3}$ represents the rotational matrix component of the pose transformation matrix $T_{n_{s},i}$.

For segment j, the generalized equivalent interaction force acting on link (j,i) $\left(1\leq j<n_{s},1\leq i\leq n_{s}\right)$ is the following:

(35) $$\begin{array}{cc} {\tilde{F}}_{j,i}^{\left(al\right)} & =A_{j,i}{\tilde{F}}_{j,i+1}^{\left(al\right)}+A_{j,i}^{\left(ci\right)}F_{j,i}^{\left(ci\right)}-F_{j,i}^{\left(dc\right)}+F_{j,i}^{\left(ext\right)}\\ & =\overset{n_{s}}{\underset{c=j}{\sum }}\overset{n_{ss}}{\underset{a=i}{\sum }}\left(\left(\prod_{b=i+1}^{a}A_{c,b-1}\right)A_{c,a}^{\left(ci\right)}F_{c,a}^{\left(ci\right)}\right)-\overset{n_{s}}{\underset{c=j}{\sum }}\overset{n_{ss}}{\underset{a=i}{\sum }}\left(\left(\prod_{b=i+1}^{a}A_{c,b-1}\right)F_{c,a}^{\left(dc\right)}\right)+\overset{n_{s}}{\underset{c=j}{\sum }}\overset{n_{ss}}{\underset{a=i}{\sum }}\left(\left(\prod_{b=i+1}^{a}A_{c,b-1}\right)F_{c,a}^{\left(ext\right)}\right) \end{array}$$

Similarly, the generalized equivalent interaction forces satisfy

(36) $$\sum_{i=1}^{n_{ss}}A_{\tau }R_{j,i}^{-1}{\tilde{F}}_{j,i}^{\left(al\right)}=0^{6\times 1}$$

This analysis demonstrates that, under ideal assumptions, the generalized equivalent interaction force is the sum of inertial force, driving cable force, and external force. Furthermore, since the generalized equivalent interaction force originates from linkage cable actions, and according to the analysis in Section 2.4.3, the linkage cable forces within a single segment cancel each other out under ideal conditions. This implies that the inertial forces, driving cable forces, and external forces of each segment must satisfy the aforementioned constraints. Consequently, since the driving cable force remains the only unknown quantity, it can be determined.

### 2.5. Target Contact Dynamics

To simplify the analysis, the target is modeled as a cylinder, where its radius corresponds to the circumscribed circle of the target. During the grasping process, when the target comes into contact with the robot, the normal contact force is given by the following:

(37) $$f_{n}=f\left(\delta \right)\cdot \left(k\cdot \delta +d\cdot \dot{\delta }\right)$$

where d is the penetration depth, k is the normal force stiffness, b is the normal force damping, s(d,w) represents a smooth function, and w is the transition region width.

The friction force at the contact point always acts opposite to the relative velocity direction at that point. Its magnitude is given by the following:

(38) $$f_{f}=\mu \cdot f_{n}$$

where μ is the friction coefficient.

## 3. Compliant Grasping Method

### 3.1. Process of Grasping

**Figure 6:**
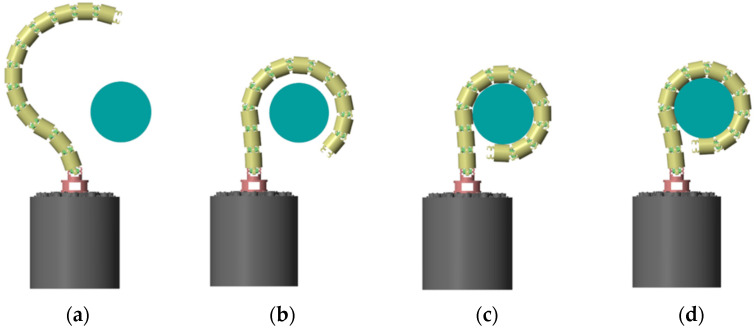
Concept of operations for WAG by CR: (**a**) State and parameter estimate phase. (**b**) Pre-grasping phase. (**c**) Grasping phase. (**d**) Post-grasping phase.

As illustrated in Figure 6, the operations for WAG using a CR can be executed in four phases. This study focuses on the grasping phase for capturing non-cooperative space targets. The CR starts from a pre-grasping configuration and follows a predetermined trajectory until a successful grasp is achieved. Firstly, a desired trajectory was generated based on the robot’s initial state and target information. Then, virtual forces were calculated using position data and, together with contact forces, were transformed into the joint space. These forces serve as inputs to an admittance controller, which calculates corrective displacements for the CR, thereby generating a new reference trajectory. The robot then moved along the updated trajectory, dynamically adapting to external forces and thereby reducing contact forces. Grasping is considered successful when the robot’s links form a stable envelope around the target and constrain its motion.

### 3.2. Virtual Force

#### 3.2.1. Virtual Repulsive Force

During WAG, virtual repulsive forces act on links that are in proximity to the target. The virtual repulsive force exerted by the target on a robot link is calculated based on position information as follows:

(39) $$F_{i}^{\left(rep\right)}=\left\{\begin{array}{cc} \begin{array}{l} f_{pchip}\left(\left\|d_{i}^{\left(bk\right)}\right\|,r_{tar}+r_{link}\right)k_{rep}\left(\frac{1}{\left\|d_{i}^{\left(bk\right)}\right\|}-\frac{1}{\rho_{rep}}\right)\frac{d_{i}^{\left(bk\right)}}{\left||d_{i}^{\left(bk\right)}|\right|},\\ 0, \end{array} & \begin{array}{l} \left\|d_{i}^{\left(bk\right)}\right\|\leq \rho_{rep}\\ \left\|d_{i}^{\left(bk\right)}\right\|>\rho_{rep} \end{array} \end{array}\right.$$

where $d_{i}^{\left(bk\right)}=P_{i}^{\left(bk\right)}-O_{tar}$, $P_{i}^{\left(bk\right)}$ is the position of points sampled along the robot’s backbone, $O_{tar}$ is the position of the target, $\rho_{rep}$ represents the effective range of the repulsive potential field, $k_{rep}$ represents the repulsive force coefficient, and $f_{pchip}$ is a smooth function related to the distance between the robot and the target.

Generally, the repulsive force increases as the distance between the robot’s link and the target decreases. In WAG tasks, insufficient virtual repulsive force may result in inadequate deceleration during approach, whereas excessive repulsive force may prevent the robot from making contact with the target. To address this, the smoothing function $f_{pchip}$ was incorporated to ensure that, when the robot link is far from the target, the repulsive force behaves normally, and, as the distance decreases, the repulsive force gradually reduces to zero.

The virtual repulsive force is transformed from task space to joint space using the Jacobian matrix:

(40) $$\tau^{\left(rep\right)}=\sum_{i=1}^{n}J_{x-q,i}^{T}F_{i}^{\left(rep\right)}$$

#### 3.2.2. Virtual Guiding Force

The ideal grasping configuration for WAG is formed by the distal segments of the CR, creating a stable grasp through the grasping segments while maintaining kinematic flexibility with delivery segments. While virtual repulsive forces reduce relative velocities between the robot’s links and the target upon contact, thereby decreasing contact forces, relying solely on these forces may lead to low grasping efficiency and unstable grasping configurations. To achieve stable and reliable compliant grasping, a virtual guiding force, comprising attractive and rotational components, was introduced and applied at the robot’s end, expressed as follows:

(41) $$\begin{array}{cc} F_{guide} & =F_{att}+F_{rot}\\ & =-k_{att}\frac{d_{end}^{\left(bk\right)}}{\left\|d_{end}^{\left(bk\right)}\right\|}\left\|d_{end}^{\left(bk\right)}\right\|-k_{rot}\frac{d_{end}^{\left(bk\right)}}{\left\|d_{end}^{\left(bk\right)}\right\|}\times z_{rot}\frac{1}{\left\|d_{end}^{\left(bk\right)}\right\|} \end{array}$$

where $k_{att}$ represents the attractive force coefficient, $k_{rot}$ represents the rotational force coefficient, and $z_{rot}$ represents the axis of the CR encircling the target. Unlike virtual repulsive forces, the virtual attractive force remains non-zero even after the robot contacts the target, ensuring stable grasping. The end of the CR is designed to maintain constant contact with the target, and a certain force applied to the target facilitates stabilization.

The virtual guiding force is transformed from task space to joint space using the Jacobian matrix:

(42) $$\tau^{\left(guide\right)}=J_{x-q,end}^{T}F^{\left(guide\right)}$$

### 3.3. Admittance Controller

The impedance model in joint space is the following:

(43) $$M_{a}\ddot{e}+D_{a}\dot{e}+K_{a}e=\tau_{ext}$$

where $e=q-q_{d}$ represents the deviation between the actual joint position and the desired joint position, $M_{a},D_{a},K_{a}$ are the mass, damping, and stiffness matrices in the impedance model, respectively, and $\tau_{ext}$ is the external torque converted to joint space.

Based on the steady-state force tracking error analysis of the control system [47], force tracking can be theoretically realized by setting the stiffness coefficient to zero. Meanwhile, the CR in this study aims to achieve WAG without requiring precise trajectory tracking during the grasping process. Thus, the impedance model used in this study is modified as follows:

(44) $$M_{a}\ddot{e}+D_{a}\dot{e}=\tau_{ext}$$

where $\tau_{ext}$ comprises two components, the virtual torque $\tau^{\left(vir\right)}$, $\tau^{\left(vir\right)}=\tau^{\left(rep\right)}+\tau^{\left(guide\right)}$, and the external torque $\tau^{\left(con\right)}$, $\tau^{\left(con\right)}=J^{T}F^{\left(ext\right)}$.

From the above equation, $\ddot{e}$ can be obtained, allowing the reference trajectory $q_{r}$ to be derived as follows:

(45) $$\left\{\begin{array}{l} {\ddot{q}}_{r}={\ddot{q}}_{d}+\ddot{e}\\ {\dot{q}}_{r}={\dot{q}}_{d}+\int \ddot{e}\mathrm{dt}\\ q_{r}={\dot{q}}_{d}+\int \int \ddot{e}\mathrm{dt} \end{array}\right.$$

**Figure 7:**
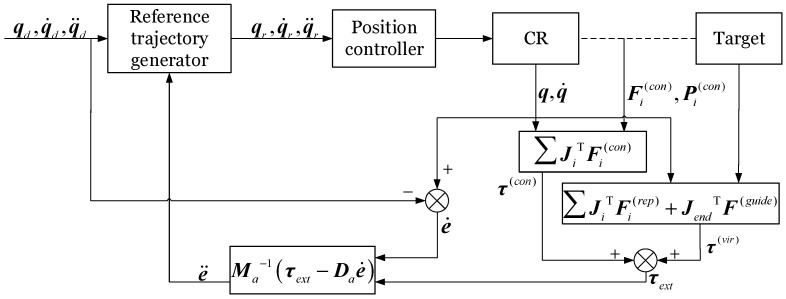
Control diagram of the VIAC method.

The control block diagram of the VIAC method is illustrated in Figure 7.

### 3.4. Position Control

Through the VIAC method, a new reference trajectory was generated. By controlling the driving cables, the CR was driven to move along the reference trajectory. The driving cable tensions were computed using the computed torque control method, which integrates feedback linearization with the robot’s dynamic model via a PD controller. The angular acceleration is calculated as follows:

(46) $$\ddot{q}={\ddot{q}}_{r}+K_{d}\dot{e}+K_{p}e$$

where $K_{p}$ and $K_{d}$ are diagonal positive-definite matrices representing the proportional and derivative gains, respectively. Here, $e=q_{r}-q$, with the reference trajectory serving as the desired trajectory. The computed joint angular acceleration was then incorporated into the inertial force calculation.

Based on the discussion in Section 2.4.4, the driving cable tensions must satisfy the constraint that the sum of the equivalent interaction forces within a single segment is zero, and the cable tensions cannot be negative. Excessive variations in cable tensions may lead to over-tensioning or slackening, negatively affecting system accuracy and stability while shortening hardware lifespan. To minimize these tension fluctuations, the driving cable tensions can be uniquely determined by solving an optimization problem:

(47) $$\left\{\begin{array}{c} \mathrm{minimize}\;F_{t}=\sum_{k=1}^{3}{\left(f_{n_{s},k}^{\left(dc\right)}-f_{n_{s},k,old}^{\left(dc\right)}\right)}^{2}\\ \mathrm{subject}\;\mathrm{to}\;\left\{\begin{array}{l} {\tilde{F}}_{n_{s},i}^{\left(al\right)}=\sum_{j=i}^{n_{ss}}\left(\left(\prod_{m=i+1}^{j}A_{n_{s},m-1}\right)A_{n_{s},j}^{\left(ci\right)}F_{n_{s},j}^{\left(ci\right)}\right)+\prod_{j=i}^{n_{ss}}A_{n_{s},j}F_{ext}\\ \;\;-\sum_{j=i}^{n_{ss}}\left(\left(\overset{j}{\underset{m=i+1}{\prod }}A_{n_{s},m-1}\right)F_{n_{s},j}^{\left(dc\right)}\right)(1\leq i\leq n_{ss})\\ \sum_{i=1}^{n_{ss}}A_{\tau }R_{n_{s},n_{ss}}^{-1}{\tilde{F}}_{n_{s},i}^{\left(al\right)}=0^{6\times 1}\\ f_{n,k}^{\left(dc\right)}\geq f_{min}^{\left(dc\right)}(k=1,2,3) \end{array}\right. \end{array}\right.$$

## 4. Simulation and Discussion

To verify the effectiveness of the proposed method, this section presents simulation validation of the VIAC method for capturing non-cooperative targets with a CR. It is important to clarify the following considerations: (1) This study focuses specifically on the grasping process and addresses the compliant control problem when the robot initiates contact with the target and forms a stable grasping configuration. Force equilibrium after grasping is beyond the scope of this paper and is not further explored. Once the grasping task is completed, the CR maintains its current configuration without additional movement. (2) External force measurement remains one of the challenges in compliant control of CRs. Existing studies have explored indirect measurement methods [46,52] and flexible skin sensors [53,54], indicating that external force measurement is technically feasible. The simulation is conducted in MATLAB/Simulink. To ensure the focus remains on the compliant grasping process, measurement data—including the kinematic states of the CR and the target, as well as contact forces exerted on the robot’s links—are obtained through dedicated measurement modules within the simulation environment.

### 4.1. Simulation Settings

The parameters of the target and the CR are shown in Table 1 and Table 2, respectively.

In practical operation, the completion of successful WAG can be determined when joint angles stabilize [38]. However, in simulation, numerical variations are subtle, making such a determination more difficult. To address this, the criteria for the completion of successful WAG in simulation are defined based on measurement data: (1) the polygon formed by all contact points must enclose the target’s centroid for at least three consecutive simulation steps, ensuring stable contact between the robot and target [55]; (2) the distance between the starting and ending points of the grasping segments must remain nearly constant over five consecutive simulation steps, indicating that the robot has formed a stable grasping configuration around the target.

### 4.2. Simulation Experiment 1

To evaluate the effectiveness of the VIAC method, two sets of compliant control parameters are selected, and kinematic and dynamic simulations are performed for the compliant grasping process of a stationary target using both admittance control (AC) and VIAC.

First, kinematic simulations are conducted, where joint angles—serving as the output of the position controller—are directly applied to control the CR. Under this ideal position control framework, position tracking errors are eliminated, allowing focus solely on how different compliant control methods affect simulation results. Specifically, the key performance metrics used to evaluate different compliant control methods include the contact force exerted on the target, the angular velocities of the CR, and the target’s velocity.

Then, dynamic simulations are conducted, where driving cable forces are computed using the computed torque control and serve as the output of the position controller. The driving cable forces are modeled as point loads applied at the locations of the cable holes, ensuring a realistic representation of physical interactions. These simulations consider actual physical effects during dynamic interaction, allowing for a comparative analysis of various compliant control methods based on the contact forces exerted on the target, the angular velocities of the CR, the target’s velocity, and driving cable tensions. The control parameters utilized in this simulation are listed in Table 3.

#### 4.2.1. Comparison and Analysis of Kinematic Simulation Results

**Figure 8:**
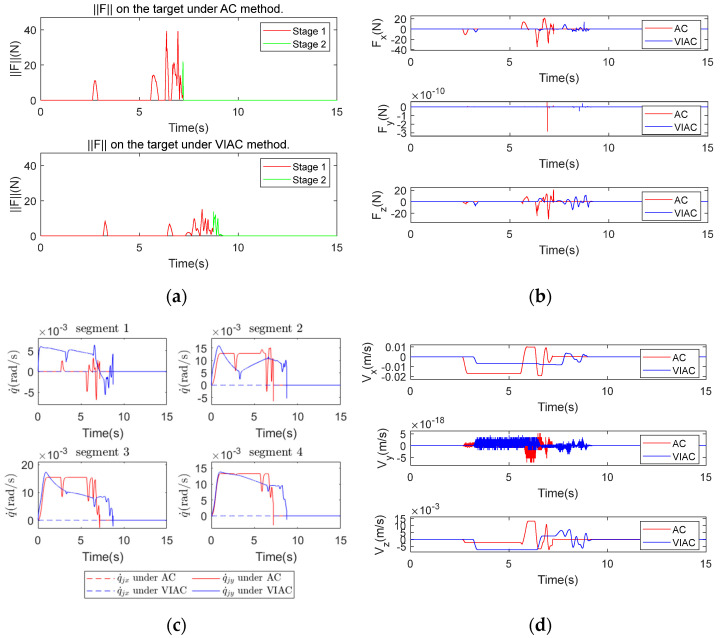
Comparison of kinematic simulation results under CP1: (**a**) contact force magnitude exerted on the target; (**b**) contact force on the target in x−, y−, z−directions; (**c**) angular velocities of the CR; and (**d**) target velocity.

Figure 8 illustrates the comparison of simulation results between AC and VIAC under CP1. Figure 8a shows the contact force magnitude exerted on the target, where force variations are categorized into two stages based on the successful grasping criteria: Stage 1 represents the grasping phase (indicated by a red solid line), while Stage 2 corresponds to the configuration holding phase (indicated by a green solid line). Figure 8b shows the variations in the contact force in three spatial directions, Figure 8c shows the angular velocities of the CR, and Figure 8d shows the changes in target velocity.

As shown in Figure 8a,b, the peak contact force is lower under the VIAC method. This reduction occurs because the virtual repulsive forces exerted on the robot links enable them to decelerate before contact with the target, thereby decreasing the relative velocities at the moment of contact and consequently diminishing the contact force on the target. However, this method leads to a longer grasping time. Since the WAG approach is employed, the velocities of multiple points along the backbone of the CR decrease as they approach the target, leading to an extended time required to achieve successful grasping. Although the VIAC method increases grasping time due to its pre-contact deceleration behavior, this trade-off is critical for space applications where contact safety is paramount. The structural fragility of non-cooperative targets, coupled with the significantly reduced damping effects in microgravity, necessitates lower contact forces, even if this results in a longer grasping time.

Figure 8c indicates that angular velocities of the CR are smoother and exhibit smaller fluctuations under the VIAC method, suggesting that the speed variations in the driving cables are also more stable. Nonetheless, sharp changes in joint velocities are observed at the moment of successful grasping confirmation for both control methods. This abrupt change results from the command to maintain the grasping configuration after successful grasping, which rapidly stabilizes the target but generates an impact force between the target and the robot, as seen in Stage 2 of Figure 8a. Despite this, the VIAC method effectively reduces contact force and enhances the compliance of the grasping process.

Figure 8d reveals that the target velocity fluctuates over a wider range under AC. According to the momentum theorem P=mΔv=FΔt, a greater change in velocity correlates with a larger change in the momentum of the target. CP1 leads the CR to demonstrate high stiffness characteristics and shorter contact duration, resulting in a greater impact transmitted to the robot.

**Figure 9:**
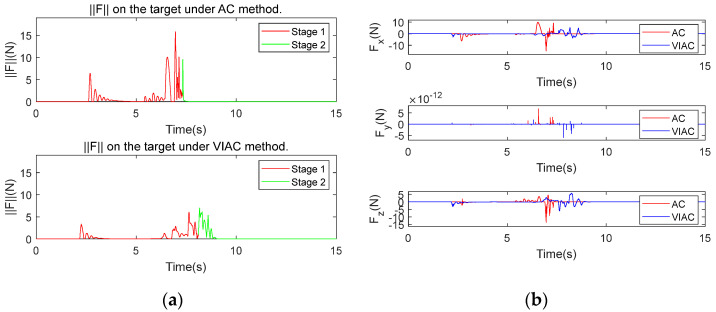

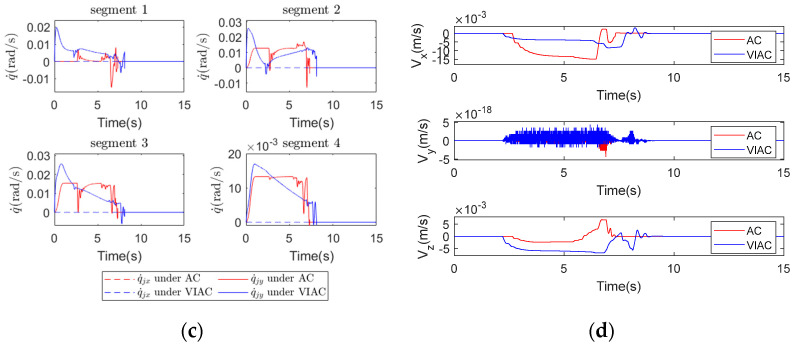
Comparison of kinematic simulation results under CP2: (**a**) contact force magnitude exerted on the target; (**b**) contact force on the target in x−, y−, z−directions; (**c**) angular velocities of the CR; and (**d**) target velocity.

Figure 9 illustrates the comparison of simulation results between AC and VIAC under CP2. Figure 9a shows the contact force magnitude exerted on the target, Figure 9b shows the variations in the contact force in three spatial directions, Figure 9c shows the angular velocities of the CR, and Figure 9d shows the changes in target velocity.

As shown in Figure 9a,b, the peak contact force is lower, and the grasping time is longer under the VIAC method. Figure 9c indicates that angular velocities of the CR are smoother and exhibit smaller fluctuations under the VIAC method. Additionally, Figure 9d shows that the target velocity fluctuates over a wider range under AC. The conclusions drawn from Figure 9 are consistent with those from Figure 8, demonstrating that the VIAC method can facilitate WAG in a smooth and compliant manner.

Although these results validate the effectiveness of the VIAC method, compliant control parameters significantly influence the system’s performance. Adjusting these parameters can lead the control system to alternate between rapid response characteristics and compliant control behavior. To analyze this effect, the simulation results from the two compliant control parameter sets shown in Figure 8 and Figure 9 are compared.

Comparing Figure 8a,b and Figure 9a,b, the peak contact forces obtained using CP2 are lower for both compliant control methods than those obtained with CP1. This reduction is because CP2 enables the robot to respond more swiftly to external forces and exhibit greater compliance. A comparison of Figure 8c and Figure 9c reveals that the corrective effect of the VIAC method on the CR’s angular velocities is more pronounced within the first two seconds under CP2. This is because the guiding force within the virtual forces dominates during this period, combined with the more compliant admittance control parameters, resulting in a larger trajectory correction for the CR. Comparing Figure 8d and Figure 9d, the range of target velocity fluctuations in Figure 9d is narrower, and the target maintains contact with the CR for a longer duration. This occurs because the CR responds more rapidly to external forces and undergoes larger corrective displacements under CP2. In this case, the CR exhibits behavior similar to an elephant’s trunk during grasping, conforming to the outer contour of the target. This compliant behavior facilitates smoother momentum transitions, reducing impulsive forces caused by sudden impacts. Consequently, the target velocity fluctuates in a narrower range, and the contact force is reduced.

#### 4.2.2. Comparison and Analysis of Dynamic Simulation Results

To simulate cable-driven actuation and intra-segment linkage, driving cable forces are applied to the links as point forces, and joints within the same segment synchronize their motion states through the modules of Simulink’s Belt and Cable Library.

**Figure 10:**
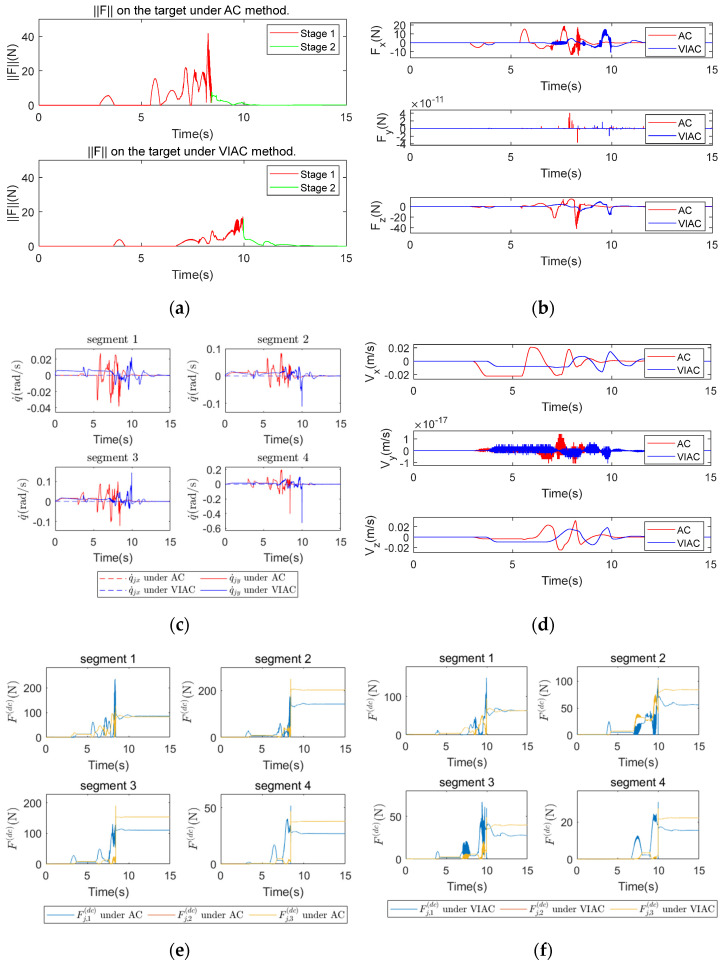
Comparison of dynamic simulation results under CP1: (**a**) contact force magnitude exerted on the target; (**b**) contact force on the target in x−, y−, z−directions; (**c**) angular velocities of the CR; (**d**) target velocity; (**e**) driving cable tensions under AC; and (**f**) driving cable tensions under VIAC.

**Figure 11:**
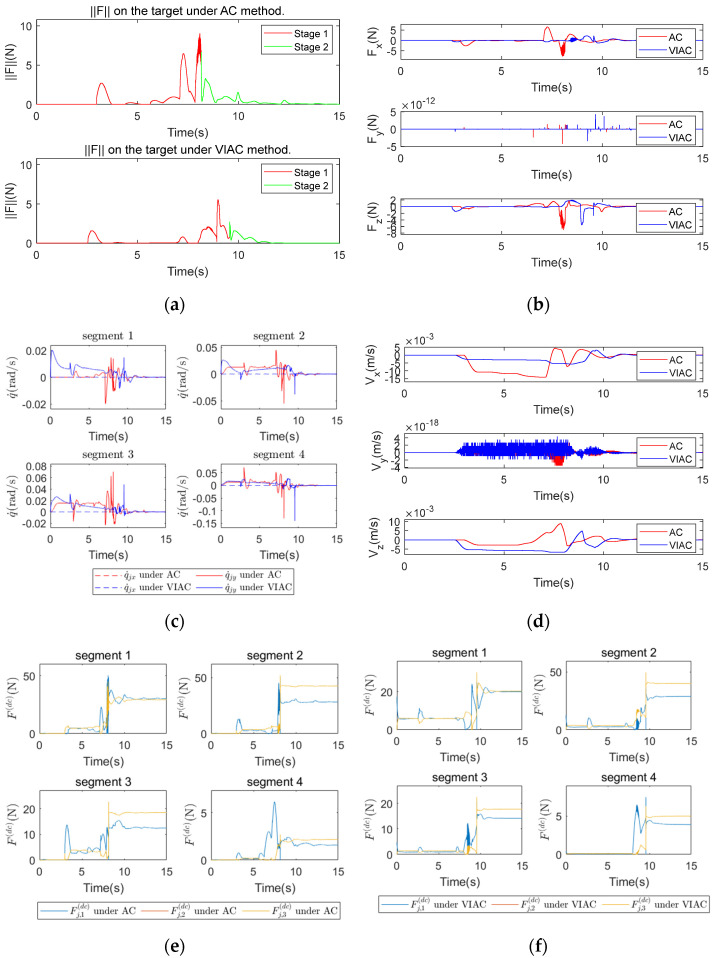
Comparison of dynamic simulation results under CP2: (**a**) contact force magnitude exerted on the target; (**b**) contact force on the target in x−, y−, and z−directions; (**c**) angular velocities of the CR; (**d**) target velocity; (**e**) driving cable tensions under AC; and (**f**) driving cable tensions under VIAC.

Figure 10 and Figure 11 present the results of the dynamic simulations. Figure 10a and Figure 11a show the contact force magnitude exerted on the target, where force variations are categorized into two stages based on the successful grasping criteria: Stage 1 represents the grasping phase (indicated by a red solid line), while Stage 2 represents the configuration holding phase (indicated by a green solid line). Figure 10b and Figure 11b show the variations in contact force in three spatial directions, Figure 10c and Figure 11c show the changes in angular velocities of the CR, and Figure 10d and Figure 11d show the changes in target velocity. Additionally, Figure 10e,f and Figure 11e,f show the driving cable tensions under AC and VIAC for CP1 and CP2, respectively.

As illustrated in Figure 10a–d and Figure 11a–d, the VIAC method consistently results in lower contact force exerted on the target, longer grasping durations, smoother angular velocity variations with smaller fluctuations, and lower target velocities under both sets of compliant control parameters. These findings align with the kinematic simulation results shown in Figure 8 and Figure 9, further confirming the effectiveness of the VIAC method in compliant grasping. However, as observed in Figure 10a, the peak contact force magnitude in Stage 2 under AC is higher than in Stage 1. This is due to the high flexibility of the CR, which results in a significant impact from the target after successful grasping. This impact causes the robot to deviate from its grasping configuration. Furthermore, the relatively high proportional gain coefficient $K_{p}$ enhances the robot’s position recovery capability, leading to greater contact forces in Stage 2.

Comparing Figure 9a and Figure 11a, the peak contact force magnitude in Figure 11a is lower than in Figure 9a due to the use of different position control strategies. Figure 9 presents kinematic simulation results, where the CR is directly controlled through joint position commands, resulting in high system stiffness. In this case, the external force can only be absorbed through trajectory adjustments in AC. Figure 11 presents dynamic simulation results, where system stiffness is defined by the PD control parameters. The synergy between the compliant control algorithm and the position control strategy allows for greater trajectory deviations to absorb external force, leading to lower contact force. Comparing Figure 10e,f with Figure 11e,f, the driving cable tensions under the VIAC method, as shown in Figure 10f and Figure 11f, are consistently lower than those under AC, as shown in Figure 10e and Figure 11e. Additionally, the driving cable tensions in Figure 11e,f are lower than those in Figure 10e,f. As derived in the dynamic modeling section, the driving cable tensions depend on both inertial forces and external forces. During the grasping process, the influence of inertial forces on the driving cable tensions is significantly smaller than that of external forces. A higher contact force between the target and the robot necessitates greater driving cable tensions, explaining the observed trends across different compliant control methods and parameter sets.

### 4.3. Simulation Experiment 2

A comprehensive discussion regarding the effectiveness of the VIAC method is presented in this section. The simulations are conducted under varying desired trajectories, target initial states, and control parameters to facilitate a systematic analysis of simulation results obtained through joint position control under different conditions.

**Figure 12:**
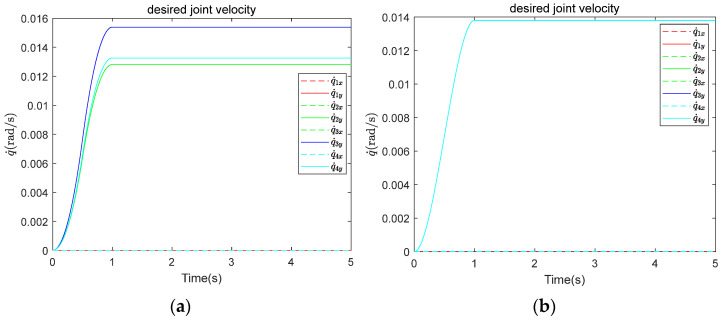
Desired trajectories: (**a**) DT1; (**b**) DT2.

Two types of desired trajectories are utilized, as illustrated in Figure 12. Figure 12a illustrates the desired trajectory 1 (DT1), in which the angular velocities of the grasping segments vary according to the planned grasping configuration. Figure 12b illustrates the desired trajectory 2 (DT2), in which the angular velocities of the grasping segments remain uniform and are independent of grasping configuration planning. To evaluate control performance in dynamic scenarios, simulations involving rotating and linearly drifting targets are conducted, in addition to those with static targets. The initial states of the target are classified as follows: (1) stationary, meaning no relative motion with the CR; (2) rotating counterclockwise around the y-axis relative to the base frame of the CR, with $\omega_{y}$ = 10°/s; and (3) moving along the x-axis relative to the base frame of the CR, with $v_{x}$ = 0.005 m/s. The variation in compliant control parameters is limited to the admittance control parameters. Using CP1 and CP2 as benchmarks, each parameter is adjusted by ±10% to create a range of values, with 20 sets of parameters randomly selected. Under the same desired trajectory and initial target state, the simulation results for these 20 sets of control parameters are compared with those obtained from the benchmark control parameters.

This study focuses on reducing the contact force exerted on the target during the grasping phase, with the recorded simulation results reflecting the peak contact force magnitude in Stage 1, rather than the entire simulation duration. Based on variations in the robot’s desired trajectories, the target’s initial states, and benchmark control parameters, a total of 12 data sets are obtained.

To evaluate the control performance under different conditions, the average maximum contact force magnitude (${\bar{F}}_{max}$), the coefficient of variation in the maximum contact force magnitude ($C_{v}(F_{max})$), and the maximum relative deviation of the maximum contact force magnitude ($\delta_{max\;}(F_{max})$) are calculated to assess the contact force levels and their fluctuations. To quantify the effectiveness of the VIAC method in reducing contact force, the average reduction in the maximum contact force magnitude for each group (${\bar{\Delta F}}_{max}$) and the coefficient of variation in the reduction in maximum contact force magnitude ($C_{v}(\Delta F_{max})$) are also computed.

**Figure 13:**
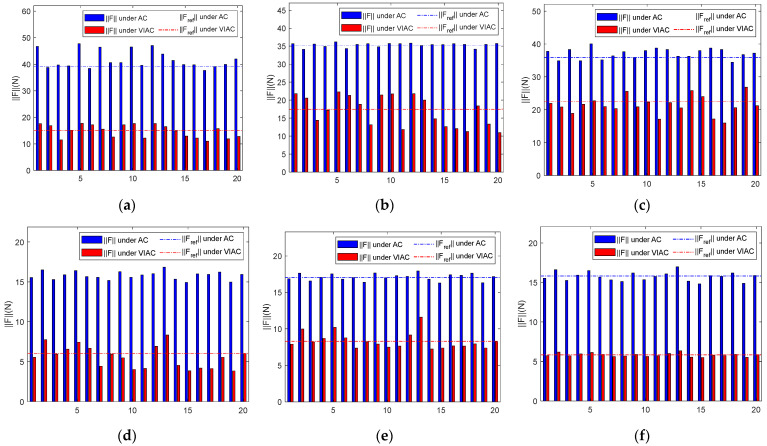
Maximum contact force magnitude under DT1: (**a**) stationary; (**b**) $\omega_{y}=10^{{}^{\circ}}/s$; (**c**) $v_{x}=0.005\;m/s$; (**d**) stationary; (**e**) $\omega_{y}=10^{{}^{\circ}}/s$; and (**f**) $v_{x}=0.005\;m/s$.

Figure 13 presents the maximum contact force magnitude obtained from simulations conducted under DT1. Figure 13a–c illustrate the maximum contact force magnitude recorded for different initial states of the target, with admittance control parameters varying based on CP1, while Figure 13d–f depict the corresponding results under CP2. Table 4 summarizes the processed data.

The results for ${\bar{F}}_{max}$ in Table 4 indicate that, despite variations in the target’s initial state and differences in the benchmark control parameter sets, ${\bar{F}}_{max}$ under the VIAC method remains consistently lower than that observed with AC, indicating the effectiveness of VIAC in the compliant grasping of realistic targets, even under parameter variations. The analysis of $C_{v}(F_{max})$ reveals that the maximum contact force magnitude obtained using AC is more stable under variations in admittance control parameters, whereas the VIAC method exhibits greater fluctuations. This suggests that, while the VIAC method effectively reduces the maximum contact force magnitude during grasping, it is more sensitive to parameter changes. Observing ${\bar{\Delta F}}_{max}$ indicates a significant reduction in contact force magnitude under the VIAC method. Comparing the results for the two different benchmark control parameter sets, $C_{v}(F_{max})$ for variations based on CP2 demonstrates less volatility than that observed for CP1 across all three initial target states. This finding indicates that compliant contact behavior enhances motion stability between the target and the robot.

**Figure 14:**
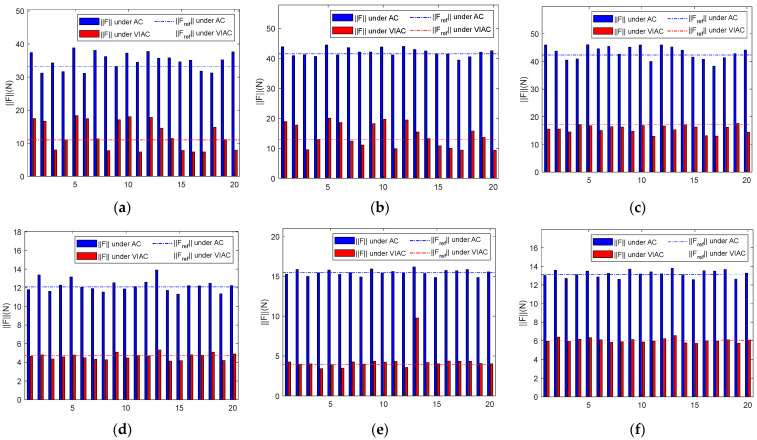
Maximum contact force magnitude under DT2: (**a**) stationary; (**b**) $\omega_{y}=10^{{}^{\circ}}/s$; (**c**) $v_{x}=0.005\;m/s$; (**d**) stationary; (**e**) $\omega_{y}=10^{{}^{\circ}}/s$; and (**f**) $v_{x}=0.005\;m/s$.

Figure 14 illustrates the maximum contact force magnitude obtained from simulations conducted under DT2. Figure 14a–c present the maximum contact force magnitude recorded for different initial states of the target, with compliant control parameters varying based on CP1, while Figure 14d–f display the corresponding results under CP2. Table 5 summarizes the processed data.

Observing the results for ${\bar{F}}_{max}$ in Table 5, it is evident that, despite the differing desired trajectories of the robot, the same conclusion can be obtained as that from Table 5. Specifically, the values of ${\bar{F}}_{max}$ under the VIAC method are consistently lower than those obtained under AC. This finding further reinforces the effectiveness of the VIAC method in reducing contact force. The results for $C_{v}(F_{max})$ indicate that values calculated under VIAC exceed those obtained from AC across all test groups, suggesting that the VIAC method exhibits greater sensitivity to control parameter variations. However, unlike the results for $C_{v}(F_{max})$ in Table 4, the comparative results under this trajectory do not exhibit consistent stability. Nonetheless, the results for ${\bar{\Delta F}}_{max}$ confirm a significant reduction in contact force under the VIAC method.

In summary, the results from Table 4 and Table 5 collectively validate the effectiveness of the VIAC method in WAG tasks for CRs. Moreover, while the VIAC method demonstrates higher sensitivity to control parameter variations, it is notably less dependent on the robot’s planned trajectory.

## 5. Conclusions

In this study, the VIAC method is proposed for CRs performing WAG. Initially, a mathematical model of a cable-driven CR is established, and, by analyzing its structural characteristics, the inverse dynamic solution problem is addressed. A compliant control method for WAG is then developed, incorporating virtual forces designed specifically for the grasping process. Both the virtual force and the contact force are simultaneously input into the admittance controller, enabling smoother interactions between the CR and the target while reducing the contact force during grasping.

To validate the efficacy of the proposed method, simulation experiments are conducted. The results indicate that, compared to AC, the VIAC method significantly reduces the peak value of contact force between the robot and the target, facilitates a smoother grasping trajectory, and decreases fluctuations in driving cable forces. Furthermore, the feasibility of VIAC is demonstrated in addressing dynamic targets under parameter variations, highlighting its effectiveness in realistic scenarios. However, the abrupt cessation of joint motion upon successful grasping may generate a larger impact force, suggesting that the post-grasping control strategy requires further refinement. Additionally, while the VIAC method remains effective across different scenarios, it exhibits higher sensitivity to control parameter variations compared to AC.

Despite demonstrating the effectiveness of the VIAC method, several areas require further investigation. First, the implementation of physical experiments remains challenging due to the lack of microgravity test platforms and high-precision external force sensors. Specifically, practical force sensing in CRs faces challenges such as calibration drift, signal delay, and difficulties in integrating sensors with deformable structures. Future research should explore hardware-in-the-loop simulation approaches and analyze how sensor inaccuracies and delays influence overall system behavior. Second, the post-grasping motion control strategy of the CR should be further optimized to ensure that both the target and the robot decelerate gradually, thereby improving system stability. Finally, as this study employs constant control parameters, future work should investigate adaptive parameter adjustment strategies to enhance system robustness.

## Figures and Tables

**Table 1:** Target parameters in simulation.

Parameter Name	Value
Geometric shape	Cylinder
Radius (m)	0.1325
Height (m)	0.2
Mass (kg)	110.309
$\mathrm{Principal}\;\mathrm{axis}\;\mathrm{inertia}\;(kg\cdot m^{2})$	[0.852, 0.852, 0.968]
Position (m)	[0.2, 0, 0.35]

**Table 2:** CR parameters in simulation.

Parameter Name	Quality	Main Dimensions	Mass (kg)	Principal Axis Inertia $\left(kg\cdot m^{2}\right)$
Base	1	Diameter 190 mm	85.510	[2.3121,2.3121,1.7993]
Hooke joint	12	Length 20 mm	0.045	$\left[0.6603,0.6603,0.9853\right]\times 10^{-5}$
Rigid link	12	Length 94 mm	0.150	$\left[0.2247,0.2247,0.1096\right]\times 10^{-3}$

**Table 3:** Compliant control parameters.

Parameter Name		Value
CP1	AC parameters	$M_{d}=50I$ $,\;D_{d}=700I$
	Virtual force coefficients	$k_{rep}=1.5$ $,\;k_{att}=2$ $,\;k_{rot}=1$
CP2	AC parameters	$\;M_{d}=10I$ $,\;D_{d}=140I$
	Virtual force coefficients	$k_{rep}=0.75$ $,\;k_{att}=0.75$ $,\;k_{rot}=0.75$
Position control parameters		$K_{p}=1500I$ $,\;K_{d}=250I$

**Table 4:** Statistics of maximum contact force magnitude under DT1.

Parameter Benchmark		Stationary		$\omega_{y}=10^{{}^{\circ}}/s$		$v_{x}=0.005\;m/s$	
		AC	VIAC	AC	VIAC	AC	VIAC
CP1	${\bar{F}}_{max}(N)$	41.7439	14.8340	35.3336	16.9801	37.0986	21.3826
	$C_{v}(F_{max})$	0.0795	0.1640	0.0163	0.2493	0.0414	0.1322
	$\delta_{max\;}(F_{max})$(%)	21.77	27.08	2.99	37.22	11.57	28.88
	${\bar{\Delta F}}_{max}$ (%)	64.51		51.88		42.22	
	$C_{v}(\Delta F_{max})$	0.0770		0.2350		0.1942	
CP2	${\bar{F}}_{max}(N)$	15.7919	5.5727	17.0967	8.3274	15.7424	5.8160
	$C_{v}(F_{max})$	0.0323	0.2491	0.0278	0.1368	0.0370	0.0389
	$\delta_{max\;}(F_{max})$(%)	6.56	38.07	5.21	39.94	7.40	8.75
	${\bar{\Delta F}}_{max}$ (%)	64.85		51.35		63.05	
	$C_{v}(\Delta F_{max})$	0.1234		0.1143		0.0071	

**Table 5:** Statistics of maximum contact force magnitude under DT2.

Parameter Benchmark		Stationary		$\omega_{y}=10^{{}^{\circ}}/s$		$v_{x}=0.005\;m/s$	
		AC	VIAC	AC	VIAC	AC	VIAC
CP1	${\bar{F}}_{max}(N)$	34.9408	12.5451	42.0974	14.3157	43.2579	15.5694
	$C_{v}(F_{max})$	0.0721	0.3482	0.0322	0.2765	0.0549	0.0909
	$\delta_{max\;}(F_{max})$(%)	16.97	66.09	7.05	54.85	9.53	24.24
	${\bar{\Delta F}}_{max}$ (%)	63.96		66.11		63.99	
	$C_{v}(\Delta F_{max})$	0.1990		0.1338		0.0458	
CP2	${\bar{F}}_{max}(N)$	12.2118	4.6223	15.4770	4.3394	13.2079	6.0358
	$C_{v}(F_{max})$	0.0545	0.0711	0.0242	0.3020	0.0290	0.0372
	$\delta_{max\;}(F_{max})$(%)	14.77	12.64	4.59	148.45	5.14	8.38
	${\bar{\Delta F}}_{max}$ (%)	62.16		72.04		54.30	
	$C_{v}(\Delta F_{max})$	0.0251		0.1089		0.0233	

## Data Availability

Data are contained within this article.
